# Oracle selection provides insight into how far off practice is from Utopia in plant breeding

**DOI:** 10.3389/fpls.2023.1218665

**Published:** 2023-07-21

**Authors:** David Vanavermaete, Steven Maenhout, Jan Fostier, Bernard De Baets

**Affiliations:** ^1^ KERMIT, Department of Data Analysis and Mathematical Modelling, Ghent University, Ghent, Belgium; ^2^ Predictive Breeding, Department of Plants and Crops, Ghent University, Ghent, Belgium; ^3^ IDLab, Department of Information Technology, Ghent University - imec, Ghent, Belgium

**Keywords:** genetic gain, genetic selection, genetic variation, genetic value, oracle, scoping, plant breeding, simulation study

## Abstract

Since the introduction of genomic selection in plant breeding, high genetic gains have been realized in different plant breeding programs. Various methods based on genomic estimated breeding values (GEBVs) for selecting parental lines that maximize the genetic gain as well as methods for improving the predictive performance of genomic selection have been proposed. Unfortunately, it remains difficult to measure to what extent these methods really maximize long-term genetic values. In this study, we propose oracle selection, a hypothetical frame of mind that uses the ground truth to optimally select parents or optimize the training population in order to maximize the genetic gain in each breeding cycle. Clearly, oracle selection cannot be applied in a true breeding program, but allows for the assessment of existing parental selection and training population update methods and the evaluation of how far these methods are from the optimal utopian solution.

## 1 Introduction

Since prehistory, when man started to settle and shifted from a hunter-gatherer to a settled-agricultural lifestyle, plants have played a crucial role in the development and survival of humankind. Over time, plants have been cultivated and selected based on morphological characteristics to improve favorable traits. Initially, phenotypic information was used to guide the selection of parental lines in plant breeding. Contemporary plant breeding methods select parents based on molecular markers such as single nucleotide polymorphisms (SNPs). Based on the idea that a phenotypic trait is controlled by many quantitative trait loci (QTLs) or genes, molecular markers can serve as proxies for these QTLs, assuming that they are in strong linkage disequilibrium with at least one QTL (de Roos et al., 2008). Genomic selection exploits this strategy to predict the genome-wide estimated breeding value (GEBV) using molecular markers that are uniformly distributed over the whole genome (Meuwissen et al., 2001). It has led to an improvement of trait performance in both animal and plant breeding and a reduction in time in between breeding cycles (Hayes et al., 2009; Cros et al., 2018).

A variety of methods aiming to maximize the long-term genetic value have been proposed. This problem can be tackled by optimizing the parental selection and by improving the prediction of the GEBVs. Different methods to update the training population (TP) have been proposed (Maenhout et al., 2010; Rincent et al., 2012; Akdemir et al., 2021), but Neyhart et al. (2017) demonstrated that most methods resulted in the same long-term genetic values as long as the TP is updated on a regular basis. Recently, Akdemir et al. (2021) proposed a genetic algorithm to select an optimal TP, outperforming the aforementioned update methods.

The genetic gain can also be maximized in the short term by increasing the selection intensity to only select the most superior lines (highest GEBVs) as parents (truncation selection) (Hayes et al., 2009; VanRaden et al., 2009). This often results in the selection of closely related individuals, reducing the genetic variation of the offspring and causing a lower genetic value in the long term (Jannink, 2010). To avoid the loss of genetic variation, alternative approaches such as the *scoping* (Vanavermaete et al., 2020) and *deep scoping* methods (Vanavermaete et al., 2021) were proposed. Compared to truncation selection, by slightly decreasing the selection intensity, the mentioned methods are able to better preserve genetic variation, increase the predictive performance and maximize the long-term genetic value. Although these methods outperform other parental selection strategies such as the population merit (Lindgren and Mullin, 1997), the maximum variance total (Cervantes et al., 2016) and the HUC with bridging methods (Allier et al., 2020), it remains difficult to evaluate to what extent these breeding strategies really maximize the long-term genetic value.

Oracle selection is a hypothetical frame of mind in which a value of interest (e.g., genetic gain) is maximized using the ground truth. Therefore, oracle selection can only be used in silico, allowing for the assessment of different selection algorithms. By assuming that oracle selection represents the optimal selection, the progress of existing parental selection methods and TP update methods can be assessed. Additionally, by exploiting the insights resulting from oracle selection, new (non-oracle) selection methods could be developed in the future.

In this paper, an oracle parental selection method and an oracle TP update method are proposed. Both oracle methods are compared with existing, state-of-the-art selection methods. In the case of TP updates, each method is assessed using a simulated breeding population with a narrow as well as a broad genetic variation. Different numerical characteristics such as the genetic value, predictive performance and genetic relationship of a breeding population using different TP update methods including oracle selection will be assessed. Finally, based on these insights, new approaches for parental and TP update methods are discussed.

## 2 Materials and methods

The base population and breeding scheme in this paper are adopted from Neyhart et al. (2017). The base population is constructed from two datasets of North American barley (Hordeum *vulgare*) from the University of Minnesota (UMN) and the University of North Dakota (NDSU), counting respectively 384 and 380 six-row spring inbred lines with 1590 biallelic SNP loci.

### 2.1 Breeding scheme

**Figure 1:**
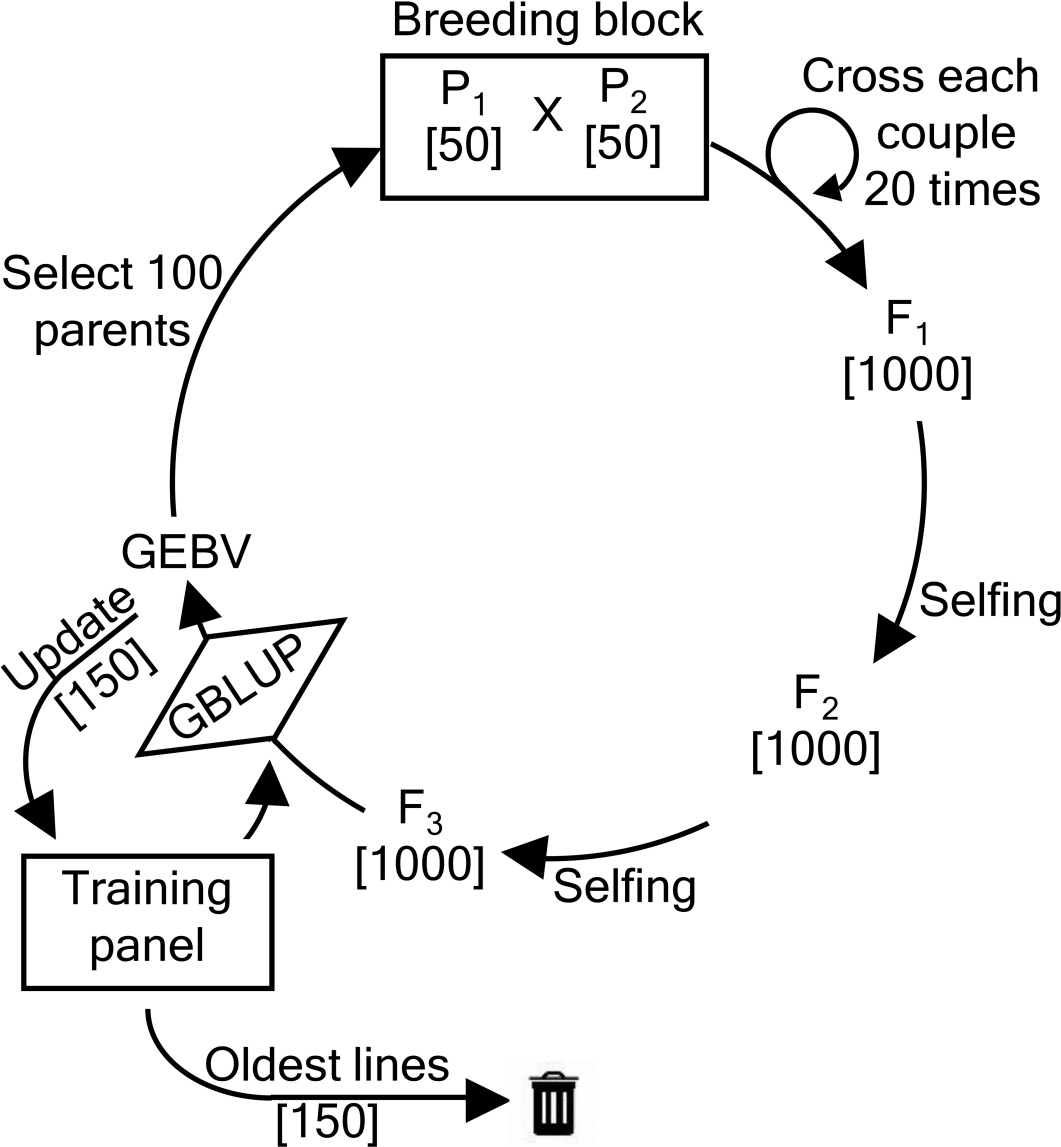
Overview of the recurrent breeding scheme. First, 50 couples of parents ( $P_{1},\;P_{2}$ ) each produce 20 offspring, yielding a total of 1000 F1 hybrids. After two generations of single-seed descent, 1000 F3 individuals are obtained. From those F3 individuals, new parental lines are selected.

The recurrent breeding scheme is depicted in **Figure 1** and has been described by Vanavermaete et al. (2020) as well as by Neyhart et al. (2017). In each breeding cycle, 100 parents are selected from the current breeding population and paired into 50 couples. Each couple produces 20 offspring resulting in a total of 1000 F1 hybrids. After two generations of single-seed descent, 1000 F3 individuals are obtained. These individuals form the new breeding population from which parents can again be selected.

In the first breeding cycle, 50 individuals with the highest phenotypic values of the NDSU dataset are coupled with 50 individuals with the highest phenotypic values of the UMN dataset. In all subsequent breeding cycles, 100 parents are selected and coupled according to one of the parental selection methods that are described further. From this point onwards, the parents are selected solely based on the genomic estimated breeding values(GEBVs). The GEBVs are predicted using a linear mixed effects model that has initially been fitted using the base population and incorporates both phenotypic and genotypic information. During each breeding cycle, the TP is updated using one of the TP update methods (described further). These methods add and remove individuals from the TP. Added individuals are selected from the current breeding population.

The different parental selection methods are compared over 50 breeding cycles, whereas the different TP update methods are compared over 15 breeding cycles. All results are averaged over 100 simulation runs.

### 2.2 Parental selection methods

Truncation selection selects 100 individuals with the highest GEBVs and crosses them randomly. This method is generally associated with a high short-term genetic gain and is therefore often used in breeding programs.

The optimal genomic mating (OGM) method, on the other hand, selects parents that minimize an objective function combining the inbreeding coefficient, the coancestry coefficient and a usefulness criterion (Akdemir and Sánchez, 2016). Parents are selected by a genetic algorithm using the R package GenomicMating.

The scoping method has been proposed by the present authors and consists of two steps (Vanavermaete et al., 2020). First, individuals with the highest GEBVs are preselected from the breeding population. The fraction of the population that is preselected is controlled by the scoping rate (SR). Second, parents are selected iteratively from the preselected individuals. The individual with the highest GEBV is selected as the first parent, whereas the second parent is the one that maximizes the F-score ( F ):


(1) $$F=\sum_{i=1}^{k}\mathrm{var}({\text{Z}}_{*,i})p_{i},$$

with k the number of markers, $Z_{i}$ the i –th column of the n×k matrix **Z** containing the genotypes (coded as -1, 0 and 1 ) of the n already selected individuals. The vector **p** consists of k Boolean values that are initially set to 1 for all marker positions. When both alleles at marker i are present, $p_{i}$ is set to 0 . If all $p_{i}$ equal 0, they are restored to 1. This way, the inclusion of all marker alleles in the parental population is assured to the highest extent possible. A complete overview of the scoping method can be found in Vanavermaete et al. (2020).

The deep scoping method uses the scoping method to (re)introduce new QTL alleles in the breeding population through the use of a genebank. First, truncation selection is used for five breeding cycles (DSBC5) to simulate a reduction in genetic variation in the breeding population due to intensive breeding. Next, a genebank is used to (re)introduce genetic variation in the breeding population. Because the genebank is characterised by a broad genetic variation and individuals with a low genetic value, the breeding population is divided into different layers allowing for the genetic values to gradually increase over different breeding cycles before introducing these individuals into the elite population. A complete overview of the deep scoping method is reported in Vanavermaete et al. (2021).

### 2.3 Oracle parental selection

Oracle parental selection is a hypothetical frame of mind that allows to reveal the full potential of parental selection. Classical selection methods only have access to information extracted from molecular markers that are linked to the QTLs that underlie the trait of interest. In contrast, oracle parental selection is allowed to exploit knowledge of the actual QTL effects. Intuitively, the method selects individuals with the highest number of favorable QTL alleles, giving priority to QTL positions that have not yet been selected in the parental population, thus preventing the loss of rare favorable QTL alleles. Formally, each individual receives a score between 0 and L (number of QTLs), representing the number of favorable QTL alleles that are present in its genome. The individual with the highest score is selected as the first parent. The remaining individuals are scored again, this time only taking into account QTL positions whose favorable alleles have not yet been selected in the parental population. In case all favorable QTL alleles are already present in at least one of the selected parents, the score is calculated over all QTLs. Again, the individual with the highest score is selected. This process is repeated until the required number of parents is selected. The selected parents are randomly paired using a recurrent breeding scheme (see **Figure 1**). The oracle selection method maximizes the genetic progress while avoiding the loss of favorable QTL alleles.

### 2.4 Training population update methods

Different methods to update the TP will be evaluated. Each method selects individuals from the current breeding population and adds them to the TP. The top TP update method selects individuals with the highest GEBVs, the tails TP update method selects individuals from both tails of the normally distributed GEBVs and the random TP update method selects individuals at random. A complete description of these methods can be found in Neyhart et al. (2017). The PEVmean TP update method selects individuals that minimize the prediction error variance, whereas the CDmean TP update method selects individuals that maximize the reliability of the predictions (Maenhout et al., 2010; Rincent et al., 2012). Finally, the TrainSel TP update method selects individuals from the breeding population by means of a genetic algorithm (Akdemir et al., 2021); this method is available in R via the package TrainSel.

### 2.5 Oracle training population updating

We propose the oracle TP update method to construct an optimal TP that maximizes the predictive performance of the genomic prediction model. The oracle TP update method is again a hypothetical frame of mind to study the characteristics of an optimally selected TP. In contrast to the oracle parental selection method, the oracle TP update method only requires the phenotypic and genotypic values of each individual. QTL information is thus not needed. The oracle TP update method can be used in combination with classical (i.e., non-oracle) parental section methods that rely on GEBVs. The difference is that by using an optimally selected TP, the GEBVs are better predicted.

In the oracle TP update method, individuals are added and removed from the TP iteratively. First, the contribution of each individual towards the predictive performance is calculated. The predictive performance is expressed as the Pearson correlation between the predicted and true genetic values of the whole breeding population. To avoid overfitting, individuals of the breeding population that have been accepted in the TP are not used to calculate the predictive performance. The individual that maximizes the predictive performance after its addition to the TP is accepted in the TP. Second, the impact of removing each individual separately from the TP is assessed and the individual that maximizes the predictive performance after its removal is eliminated from the TP. In total, up to 50 individuals can be added and removed from the TP. An individual will only be added to or removed from the TP if that action increases the predictive performance. It is, however, possible that each individual in the TP has a positive contribution towards the predictive performance and that no individual is removed. Therefore, the size of the TP could vary, depending on the addition and removal of individuals.

### 2.6 Prediction model

In the first breeding cycle, the complete base population is used as TP. In the subsequent breeding cycles, 150 individuals are phenotyped and added to the TP according to the tails method, selecting 75 individuals with the highest and 75 individuals with the lowest GEBVs (Neyhart et al., 2017). According to Neyhart et al. (2017), this results in a (non-significantly) higher genetic gain compared to other update methods. Before updating the TP, the 150 oldest individuals in the TP are removed from the TP. This reduces the computational time without reducing the predictive performance (Neyhart et al., 2017).

In the case of the oracle TP update method, only 100 randomly selected individuals of the base population are used as TP. To compare the different TP update methods, in subsequent breeding cycles, only up to 50 individuals are added to the TP. Additionally, the oracle TP update method can also remove up to 50 individuals from the TP if the removal of such individuals increases the predictive performance.

The GEBVs are predicted by fitting a linear mixed effects model:


(2) $$\text{y}=1_{n}\beta +\text{Zu}+\epsilon ,$$

with **y** a vector of phenotypic values, **1** *_n_* a vector of size n containing ones, n the number of individuals in the TP, β the fixed effect (phenotypic mean), Z he incidence matrix of the TP with marker information, u the marker effects following a normal distribution N(0,G) with $G=\sigma_{u}^{2}I_{k}$ (with $I_{k}$ the identity matrix of dimension k ), k the number of markers and ε the residual effects following a normal distribution N(0,R) with $R=\sigma_{e}^{2}I_{n}$ . Both $\sigma_{u}^{2}$ and $\sigma_{e}^{2}$ are estimated by means of restricted maximum likelihood (REML). The GEBVs of the individuals are calculated as:


(3) $$\hat{g}=Z\hat{u},$$

with $\hat{g}$ the GEBVs, Z the marker information and $\hat{\text{u}}$ the predicted marker effects.

The linear mixed effects model in Eq. (2) is fitted using the package rrBLUP in R (Endelman, 2011). Even though it has been recommended to remove markers with low levels of polymorphism from the TP (Chang et al., 2018), we kept all markers as this resulted in a higher predictive performance.

### 2.7 Simulation of the population

The simulation study was built upon the work of Neyhart et al. (2017), using the packages GSSimTPUpdate and hypred in R (version 3.6.3). First, the QTLs were simulated based on the marker position, allele, and chromosomal information. One hundred QTLs ( L=100 ) are selected randomly from the available 1590 biallelic SNP loci. The remaining 1490 biallelic SNP loci are available as markers for prediction and selection purposes. The QTL effects are calculated according to a geometric series. At the k -th QTL, the favorable homozygote will have a value $a^{k}$ , the heterozygote a value zero, and the unfavorable homozygote a value $-a^{k}$ with a=(L−1)/(L+1) . Dominance and epistatic effects were assumed to be absent. The phenotypic value is calculated over three different environments, each drawn from a normal distribution with mean 0 and variance $\sigma_{E}^{2}$ which is defined as 8 var(*g*) with g the genetic values of the breeding population (Bernardo, 2014). The variance of the genetic value, and hence $\sigma_{E}^{2}$ is calculated before the first breeding cycle and remains unchanged during the simulation. The phenotypic value of the i -th individual in the j -th environment ( $y_{ij}$ ) is calculated as follows:


(4) $$y_{ij}=g_{i}+e_{j}+\epsilon_{ij},$$

with $g_{i}$ the genetic value of the i -th individual, $e_{j}$ the j -th environmental effect and $\epsilon_{ij}$ the residual effect of the i -th individual and the j -th environment. The residual effect is drawn from a normal distribution with mean 0 and variance $\sigma_{R}^{2}$ , with $\sigma_{R}^{2}$ scaled to simulate a population with a heritability $\left(h^{2}\right)$ of 0.5. The residual error $\sigma_{R}^{2}$ is calculated as:


(5) $$\sigma_{R}^{2}=3\left(\frac{\text{var}\left(g\right)}{h^{2}}-\text{var}\left(g\right)\right).$$

The phenotypic value of an individual is defined as the average over the three environments. A comprehensive overview of the simulation study can be found in Vanavermaete et al. (2020).

To track the fixation of unfavorable QTL alleles, the maximum reachable genetic value is calculated as the sum of the QTL effects that are fixed (both favorable and unfavorable) and the sum of the favorable QTL effects that are not yet fixed. It represents the maximum genetic value that could still be reached, taking into account the fixation of unfavorable QTL alleles. The maximum reachable genetic value and the mean genetic value are rescaled such that the maximum reachable genetic value, when no unfavorable QTLs are fixed, has a value of 1. As in Vanavermaete et al. (2020), the mean genetic value of the top-10 individuals is reported. These individuals represent the superior lines that are prime candidates for commercialization.

## 3 Results

### 3.1 Oracle parental selection

**Figure 2:**
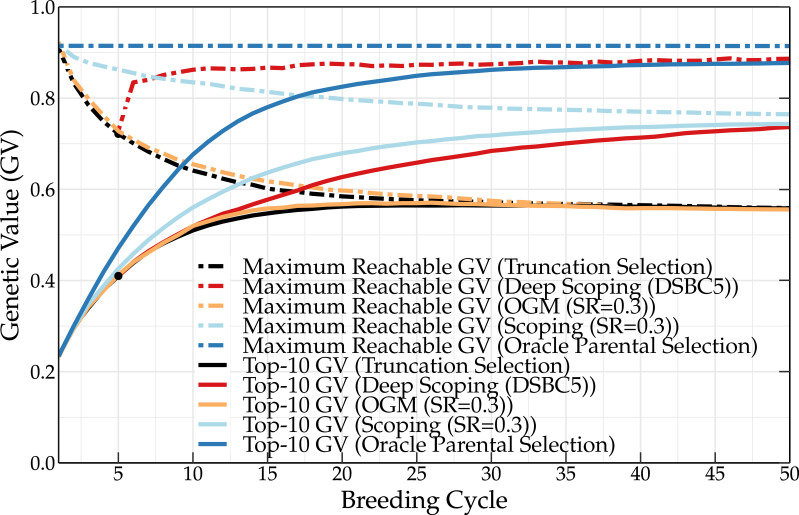
Mean genetic value of the top-10 individuals and maximum reachable genetic value of a breeding population using the oracle selection method, scoping method (SR = 0.3), and deep scoping method (DSBC5), OGM method and truncation selection over 50 breeding cycles. Oracle selection leads to a high increase of the mean genetic value over the first breeding cycles, while the maximum reachable genetic value remains constant, indicating that no favorable QTL alleles are lost. The difference in genetic value between the oracle selection method and the other methods indicates that further improvements of the parental selection methods could increase the genetic value up to 14 percentage points.

Oracle parental selection is a hypothetical frame of mind that uses the knowledge of QTL positions and QTL effects to demonstrate the effect of an almost perfect parental selection on the genetic value. In the initial population, some of the QTLs are already fixed for one of the two possible alleles. Some of these alleles have a negative contribution to the genetic value. This explains why the maximum reachable genetic value is slightly lower than 1 for the initial population (see **Figure 2**). Over the subsequent breeding cycles, the maximum reachable genetic value remains unchanged, indicating that no favorable QTL alleles are eliminated during breeding. Meanwhile, the frequency of favorable QTL alleles in the breeding population increases, leading to high mean genetic values. Over time, unfavorable QTL alleles are lost from the breeding population, leading to the fixation of favorable QTL alleles.

The scoping method (SR=0.3) and the deep scoping method (DSBC5) are able to increase the long-term genetic values compared to truncation selection (baseline) and the OGM method. However, oracle selection reaches much higher genetic values in the short as well as in the long term. This indicates that current parental selection methods could, in principle, be further optimized to increase the short- and long-term genetic values up to 14 percentage points. An overview of the mean genetic value of the top-10 individuals and of the maximum reachable genetic value for the different parental selection methods is listed in **Tables S1**, **S2** in the **Supplementary Material**, respectively.

### 3.2 Oracle training population update

The oracle TP update method is a hypothetical frame of mind that selects individuals to construct a TP using their genotypic and phenotypic values. The optimal TP is then used to (re-)train the mixed effects model that predicts the GEBVs of the current breeding population. In turn, these GEBVs are used to select parents for the next breeding cycle. Assuming that the oracle TP update method results in a good prediction of the GEBVs, it can be used to assess the current progress of other TP update methods.

**Figure 3:**
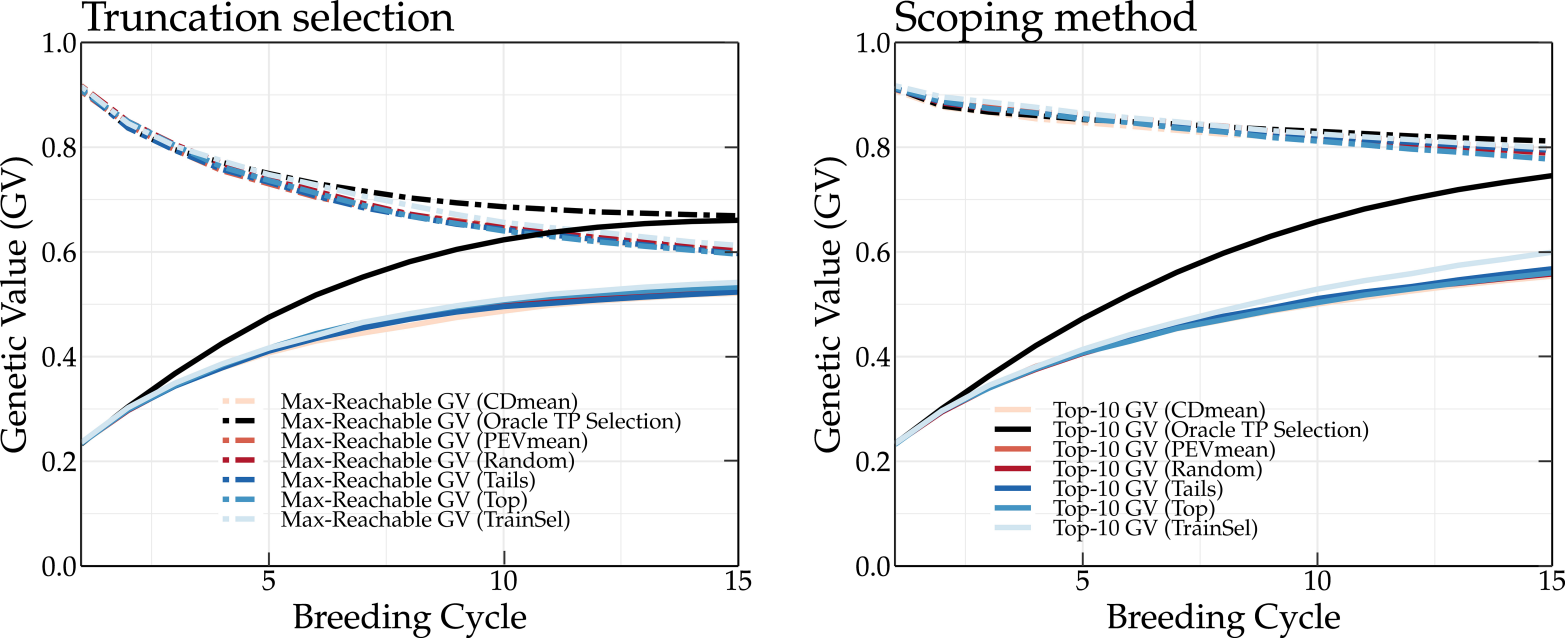
Simulation results of different TP update methods (top, tails, random, CDmean, PEVmean and TrainSel) and the oracle TP update method. The oracle TP update method yields the highest long-term genetic values.

The TP update methods were compared using a breeding population where the parents are either selected according to truncation selection or according to the scoping method. Truncation selection prioritizes individuals with the highest GEBVs, whereas the scoping method will also select individuals that maximize the genetic variation of the parental population. Therefore, both approaches may require a different TP update strategy. The results for truncation selection (left panel) and the scoping method (right panel) using different TP update methods are shown in **Figure 3**.

At the start of the simulation, each method results in the same mean genetic value of the top-10 individuals. After only two breeding cycles, the oracle TP update method already yields a higher genetic gain compared to the other TP update methods. At breeding cycle 15, a difference of 15 and 21 percentage points is observed between the oracle and top TP update methods for a breeding population using truncation selection and the scoping method, respectively. It is clear that the oracle TP update method results in a better prediction of the GEBVs, optimizing the parental selection and thus leading to higher long-term genetic values. An overview of the mean genetic value of the top-10 individuals and of the maximum reachable genetic value using different update methods is listed in **Tables S3**, **S4**, respectively, for truncation selection, and **Tables S5**, **S6**, respectively, for the scoping method.

The top, random, tails, PEVmean and CDmean TP update methods (Rincent et al., 2012; Neyhart et al., 2017) yield approximately the same genetic values. When parents are selected according to truncation selection, the top TP update method yields high genetic values, whereas when the scoping method is used, the tails TP update method results in higher long-term genetic values. The TrainSel TP update method (Akdemir et al., 2021) is able to outperform both the top and tails TP update methods in both scenarios (truncation selection and scoping method). Especially when the scoping method is used, the TrainSel TP update method results in a 3 percentage points higher genetic gain after 15 breeding cycles compared to the tails TP update method.

## 4 Discussion

### 4.1 Strength of the oracle selection methods

The oracle selection method is a theoretical concept that uses QTL positions, QTL effects, and genotypic and phenotypic information to select an optimal parental population or TP. This method allows for the comparison of currently existing methods. If a method obtains a similar genetic value as observed for the oracle method, then this means it is optimal and cannot be further improved upon. However, as observed in **Figure 2**, this is not yet the case for the current methods. Additionally, the oracle method can provide insights into which variables (e.g., the genetic relationship, inbreeding coefficient, coancestry coefficient, etc.) should be controlled to maximize the short- or long-term genetic gain.

### 4.2 The greedy selection of QTL alleles

Oracle parental selection was developed to study the effects of using a modified truncation selection scheme in which the frequency of all favorable QTL alleles is maximized. Oracle parental selection assumes knowledge of the actual QTL effects and is therefore only of conceptual interest; *in vivo*, only genetic markers are available to guide parental selection. Although oracle selection is able to maximize the genetic gain by greedily selecting the favorable QTL alleles in the parental population, when the parental selection process relies on genetic markers that are putatively linked to the causal QTL effects, greedily selecting individuals (as observed for truncation selection) often results in a premature convergence of the genetic value. In other words, by only preserving the marker alleles that have a positive estimated marker effect, the loss of favorable QTL alleles cannot be prevented. Preserving both marker alleles in the breeding population prevents the elimination of poorly estimated QTL alleles resulting in higher long-term genetic values compared to a greedy strategy like truncation selection.

### 4.3 Reaching the theoretical maximum genetic value

**Figure 4:**
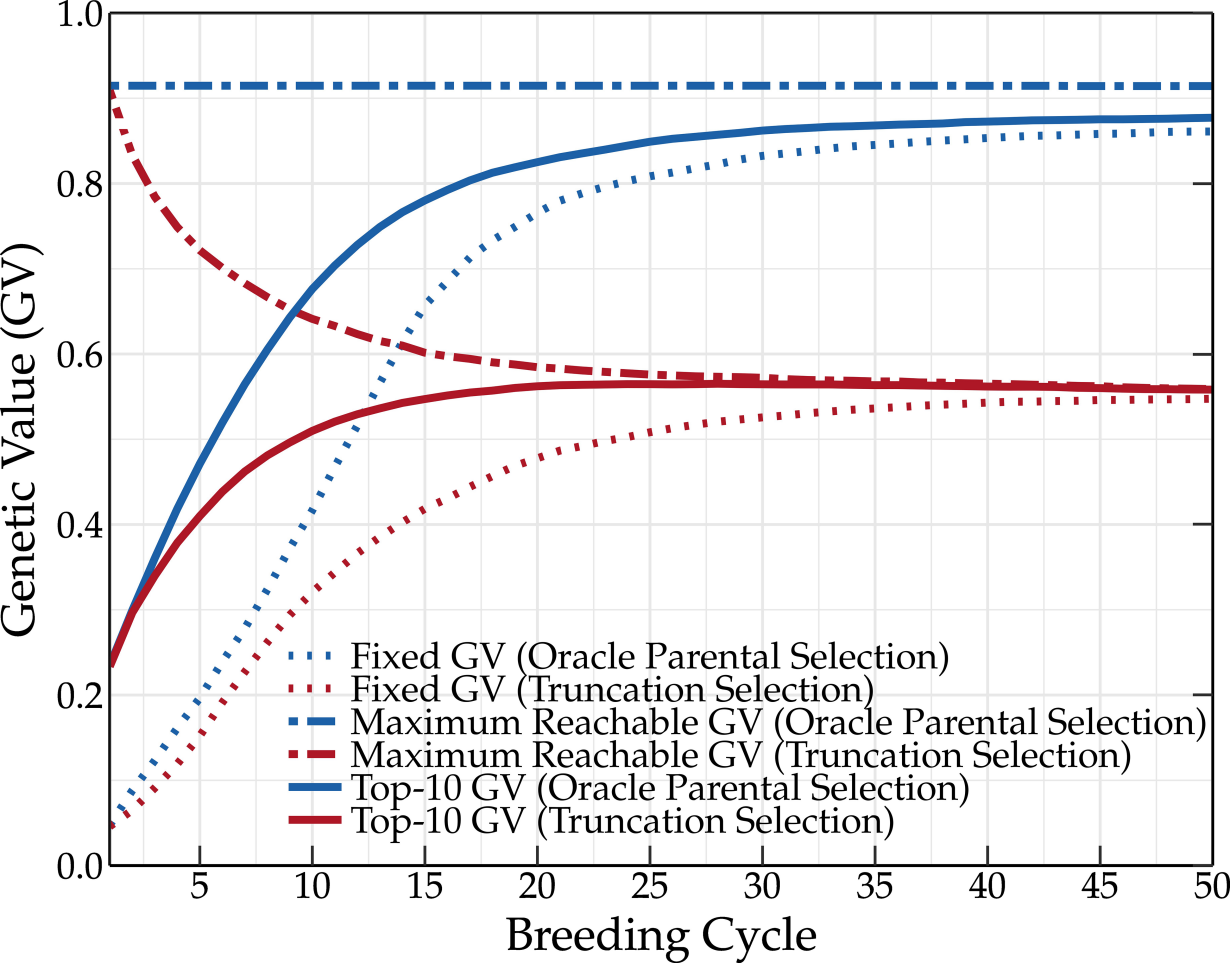
Simulation results using the oracle selection method over 50 breeding cycles. Oracle selection leads to a high increase of the mean genetic value over the first breeding cycles. The maximum reachable genetic value remains constant, indicating that no favorable QTL alleles are lost. Due to selection, the frequency of the favorable QTL alleles increases, finally leading to the loss of unfavorable QTL alleles. This eventually results in high genetic values.

The favorable QTL alleles are not always abundantly present in the initial population and many breeding cycles may be needed before fixation occurs. This explains the relatively slower increase of the fixed genetic value compared to the mean genetic value of the top-10 individuals (see **Figure 4**). In a standard setting, the fixed genetic value represents the overall effect of all QTL alleles that are fixed in the breeding population. As oracle parental selection avoids the fixation of unfavorable QTL alleles, the fixed genetic value can, in this case, be used to monitor the fixation of favorable QTL alleles. After almost 30 breeding cycles, the genetic value and the fixed genetic value converge to a slightly lower value than the maximum reachable genetic value. Oracle parental selection, which was designed to prevent the loss of favorable QTL alleles, should make it possible to reach the maximum genetic value. However, when two or more QTL alleles are in strong linkage disequilibrium (LD) w.r.t. one another, linking a favorable QTL allele to an unfavorable QTL allele, fixation of both QTL alleles becomes difficult. This was the case for five percent of the QTLs, preventing the genetic value from reaching its absolute maximum and explaining why the genetic value and the fixed genetic value did not converge to the same value. The oracle selection method demonstrates that, even in an ideal situation, at least 30 breeding cycles are needed to approach the maximum reachable genetic value in the breeding population for the base population and simulation settings used in this study.

### 4.4 Genetic values, phenotypic values, and genomic estimated breeding values

**Figure 5:**
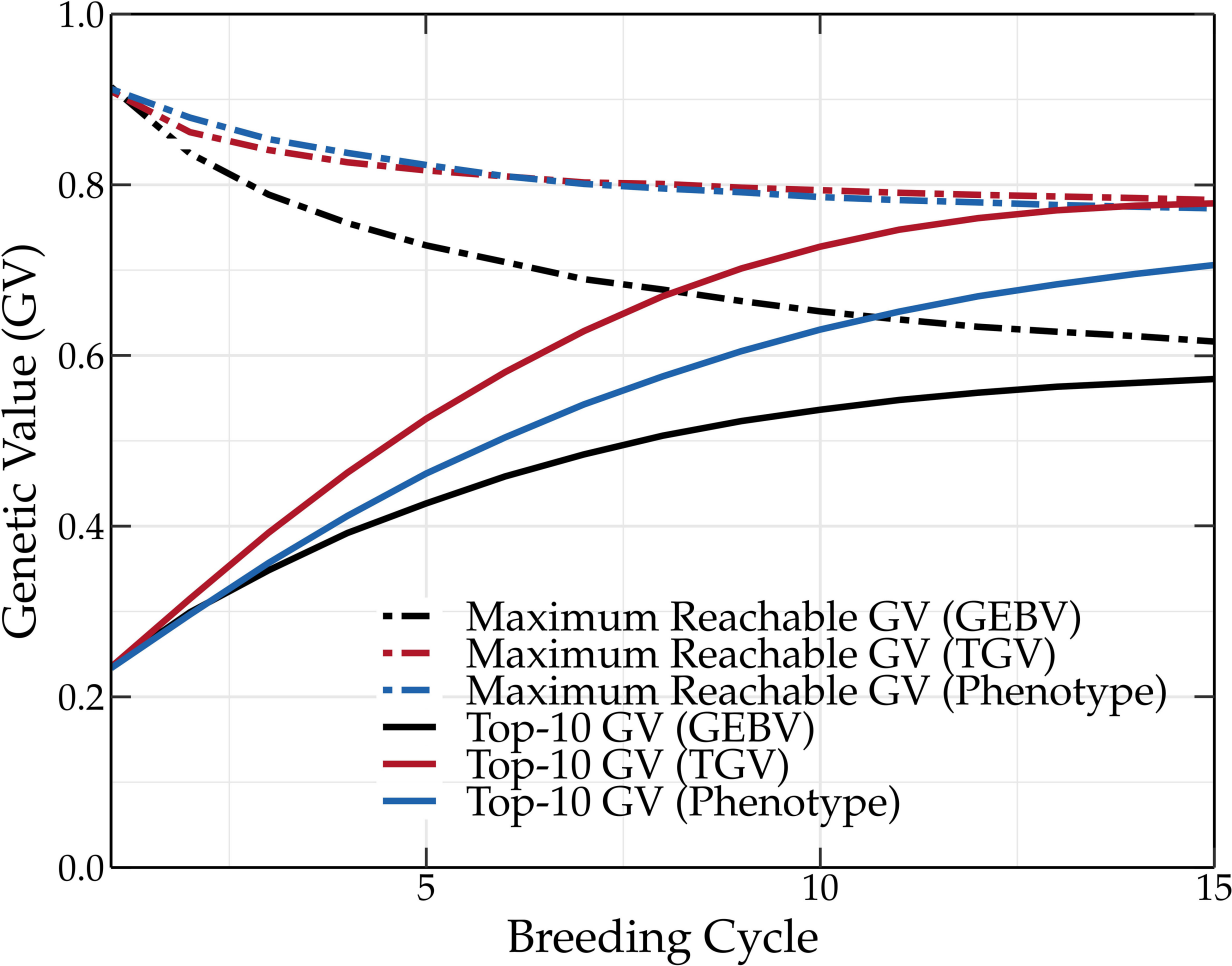
Mean genetic value of a breeding population using truncation selection. The parents are selected based on the GEBVs, true genetic values (TGVs), or phenotypic values. Selecting parents based on the TGVs results in the highest genetic values, followed by phenotypic values and GEBVs (TP updated by means of the tails method).

The main goal of breeding is to maximize the genetic value of various traits of interest and this both in the short and the long term. Unfortunately, the genetic value cannot be measured, and thus selection is often based on the phenotype. Because the phenotype is also influenced by the environment, its use to guide parental selection will result in a lower genetic gain compared to the use of genetic values. This is shown in **Figure 5**. Measuring the phenotype is a time-consuming and expensive process, therefore, GEBVs are often used instead, predicting the genetic values using a linear mixed effects model. The selection of superior individuals using GEBVs hinges on the predictive performance of the underlying genomic prediction model. The construction of a genomic prediction model is based on a TP for which phenotypic and genotypic data is required. Due to prediction errors, selecting parents based on GEBVs results in a lower genetic gain compared to parental selection based on phenotypic or genetic values (see **Figure 5**). The difference in the genetic value obtained by selecting the parents based on the GEBVs and phenotypic values could be reduced by using a more accurate prediction model and a better TP design. However, linear mixed models such as rr-BLUP and gBLUP generally achieve competitive predictive performances (Moser et al., 2009), and according to Neyhart et al. (2017), as long as the TP is updated, the genetic value converges to the same long-term value.

Aside from environmental effects and prediction errors, selecting the parents with the oracle selection method results in a higher long-term genetic value than when the parents are selected with truncation selection based on the true breeding values. Truncation selection selects individuals with the highest genetic value. Because the genetic value is calculated as the sum of all the QTL effects, an individual with a high genetic value can still possess unfavorable QTL alleles. In other words, truncation selection based on genetic values can still result in the loss of favorable QTL alleles. Oracle selection prevents, just like the (deep) scoping method, the loss of these favorable QTL alleles, resulting in an overall higher long-term genetic value.

### 4.5 Size of the training population

**Figure 6:**
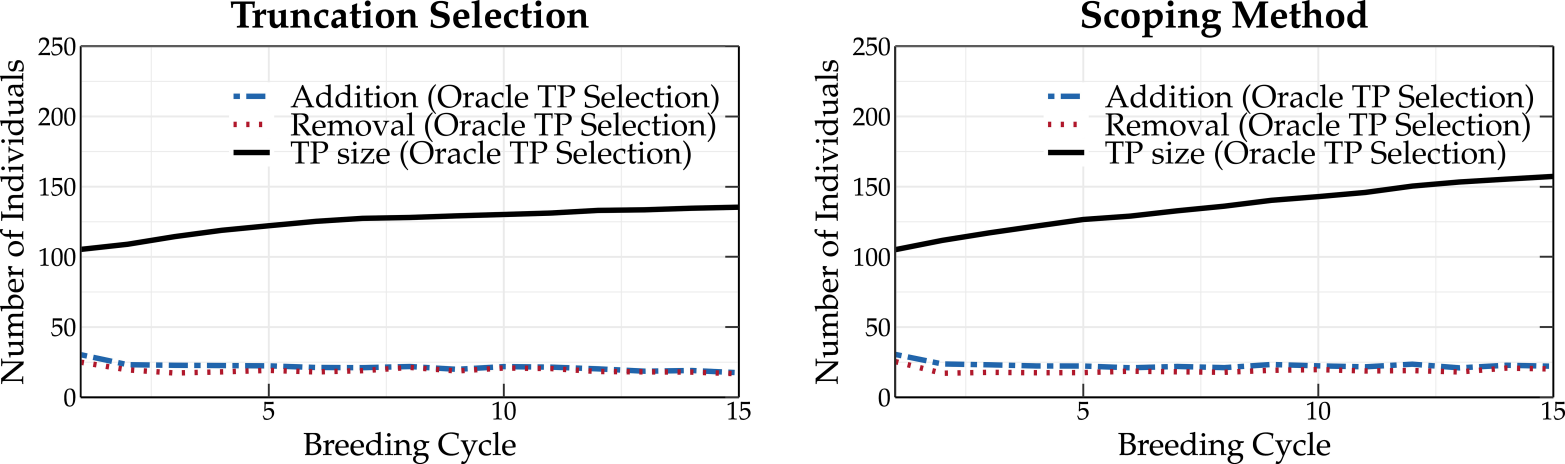
Overview of the number of individuals that are added to and removed from the TP using the oracle TP update method. Parents are selected according to truncation selection (left) or the scoping method (right). Over the first breeding cycles, a lower number of individuals are removed from the breeding population, allowing for an increase in the size of the TP.

**Figure 6** shows the size of the TP as a function of the breeding cycle when the oracle TP update method is used in combination with truncation selection (left panel) and scoping method (right panel). At the start of the simulation, the TP is constructed as a random selection of 100 individuals from the base population. The oracle TP update method can, each cycle, add and remove up to 50 individuals from the TP. An individual can only be added to or removed from the TP if a higher predictive performance is obtained as a consequence. A similar TP update pattern is observed for both truncation selection and the scoping method. However, after breeding cycle 5, the scoping method results in a slightly higher TP size compared to truncation selection. This can be explained by the inherent aim of the scoping method to increase the genetic variation in the breeding population and therefore requires a larger TP to accurately predict all GEBVs.

At the first breeding cycle, the oracle TP update method adds approximately 30 individuals to and removes 30 individuals from the TP, replacing the randomly chosen individuals one by one. In subsequent breeding cycles, the number of individuals that are removed from the TP is reduced, allowing for an increase in the size of the TP. Over time, fewer individuals are added to the TP, resulting in the same number of individuals that are added to and removed from the TP (steady state). This means that the predictive performance is not maximized by consistently increasing the TP size.

For truncation selection, the size of the TP starts to converge at breeding cycle 15. At that point, fewer than 25 individuals are added to the TP. This observation seems to indicate that using huge datasets to fit a prediction model may not be the best strategy.

### 4.6 Constructing an optimal training population

**Figure 7:**
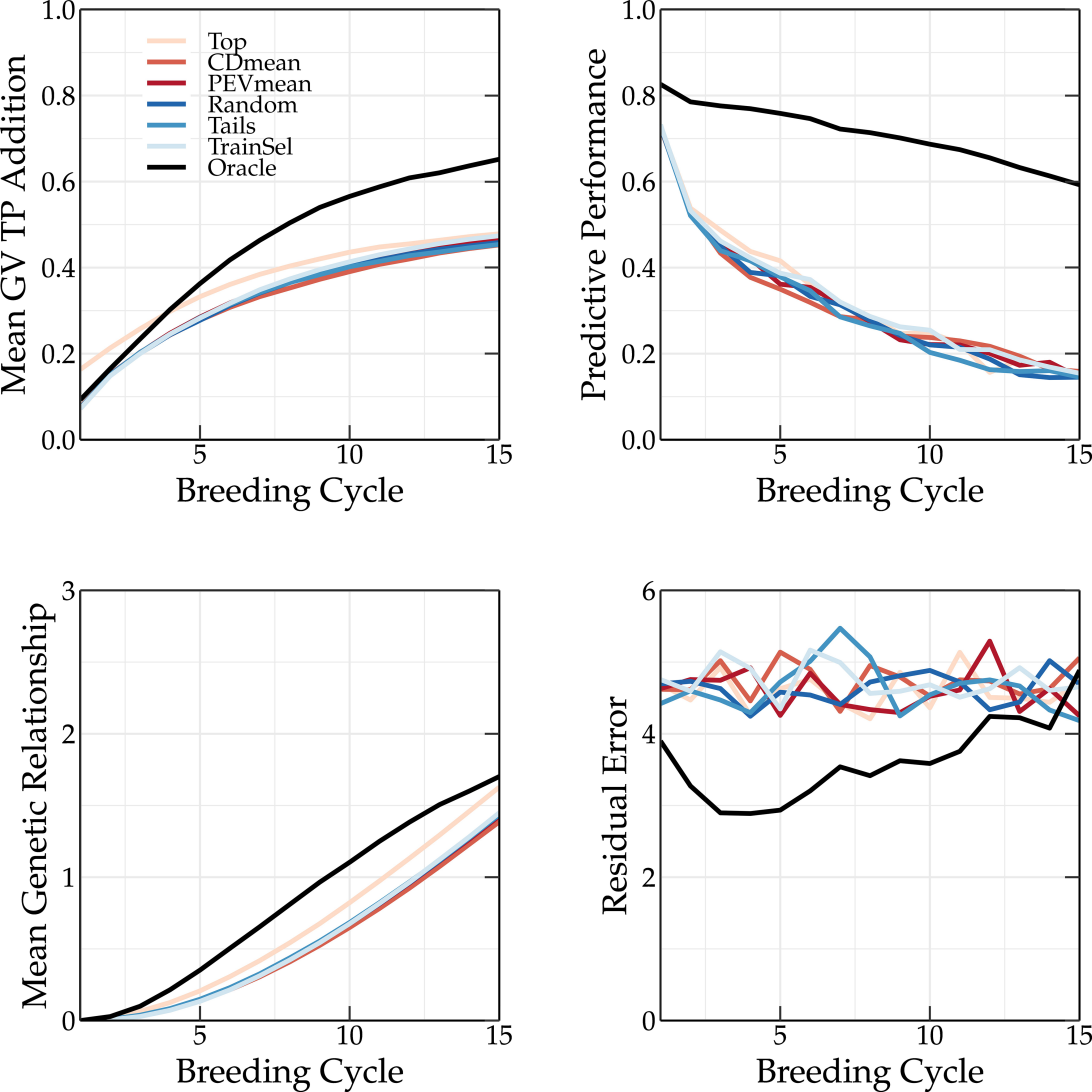
Simulation results using various TP update methods (Top, CDmean, PEVmean, Random, Tails, TrainSel, Oracle) using truncation selection to select the parents in each breeding cycle. Top left: the mean genetic value of the TP; top right: the predictive performance; bottom left: the mean genetic relationship calculated according to VanRaden (2008) between the members of the breeding population and the members of the TP scaled to the average genetic relationship between the members of the base population; and bottom right: the absolute residual error.

**Figure 8:**
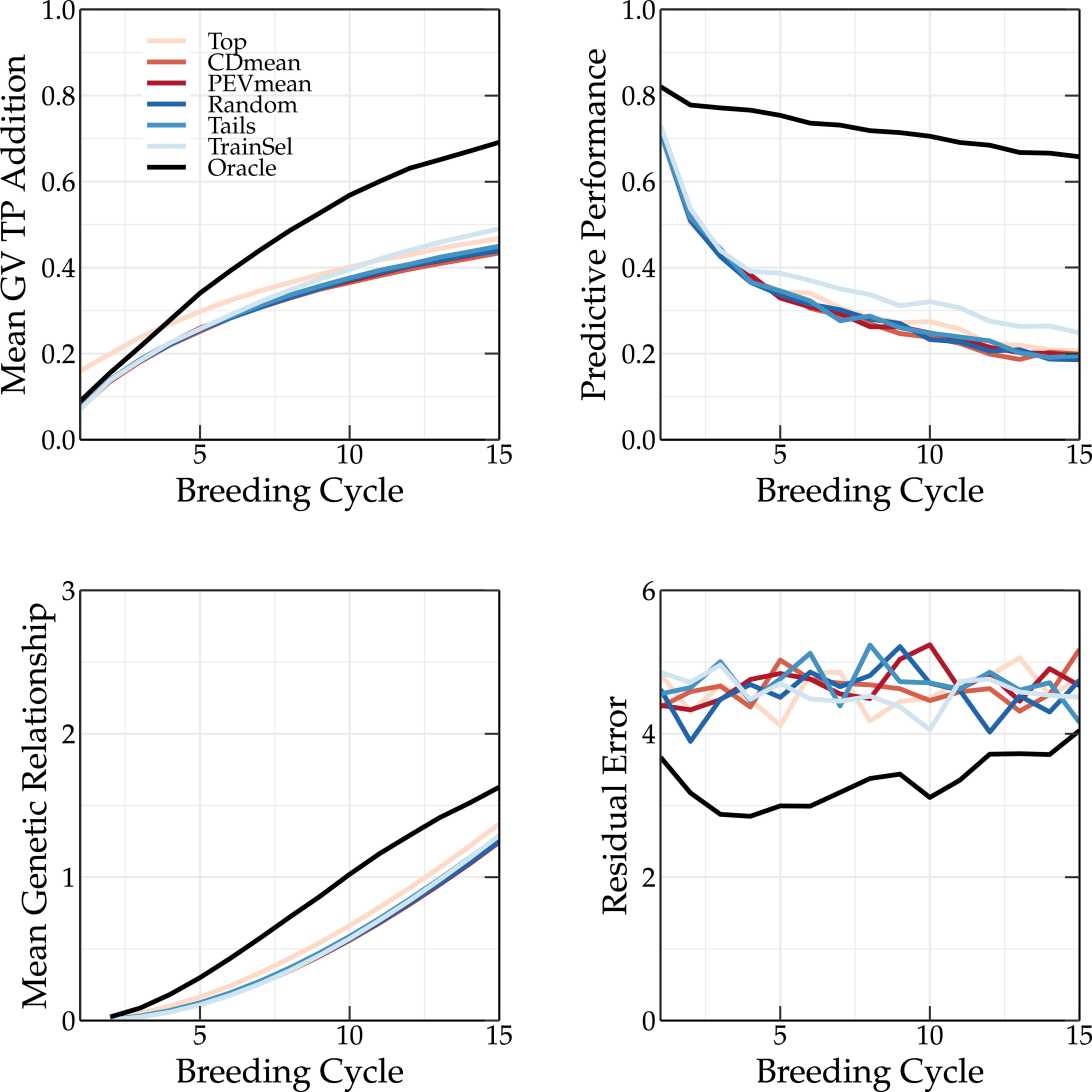
Simulation results using various TP update methods (Top, CDmean, PEVmean, Random, Tails, TrainSel, Oracle) using the scoping method to select the parents in each breeding cycle. Top left: the mean genetic value of the TP; top right: the predictive performance; bottom left: the mean genetic relationship calculated according to VanRaden (2008) between the members of the breeding population and the members of the TP scaled to the average genetic relationship between the members of the base population; and bottom right: the absolute residual error.

The top and tails TP update methods rely on GEBVs. Because the top TP update method selects only the individuals with the highest GEBVs, the mean genetic value of the TP is higher compared to that of the breeding population (see top left panel of **Figures 7**, **8**). This is not observed for the oracle TP update method. Selecting on the basis of the highest GEBVs does not seem like the best strategy to maximize the predictive performance of the GEBVs.

The oracle TP update method yields a much higher predictive performance than the other methods, whose performances are similar (see top right panel of **Figures 7**, **8**). As discussed in Vanavermaete et al. (2020), by preserving the genetic variation in the breeding population, an overall higher Pearson correlation coefficient between the true and predicted breeding values is obtained. This can be observed in **Figures 7** and **8**.

A TP should contain individuals that represent the genetic diversity of the current breeding population, allowing for a better prediction of each individual. This is also observed in **Figure 3**, where the tails TP update method results in higher long-term genetic values compared to the top TP update method. The scoping method maximizes the genetic variation, therefore, only selecting individuals with high GEBVs will not be sufficient to accurately predict the GEBVs of the whole breeding population. If individuals of the TP are a good representation of the breeding population, both will have a high genetic relationship. This can easily be calculated as:


(6) G=MM'2∑i=1kPi(1−Pi),

with **M** a matrix with n rows and k columns of which each column is calculated as $Z_{i}-1_{n}\left[2\left(P_{i}-0.5\right)\right]$ , n the number of individuals in the breeding population, $Z_{i}$ the genotype of n individuals at the i -th marker, $1_{n}$ a vector of size n containing ones, k the number of markers, and $P_{i}$ the frequency of the alternative allele at the i -th marker (VanRaden, 2008).

The mean genetic relationship between the members of the TP and the members of the breeding population for different TP update methods is shown in the bottom left corner of **Figures 7** and **8** for truncation selection and the scoping method, respectively. In the top TP update method, the individuals selected as parents are also added to the TP, resulting in a slightly higher genetic relationship between the TP and the breeding population compared to other similar TP update methods (random, PEVmean, CDmean and tails). This difference is more pronounced when truncation selection is used, since in the scoping method only the first parent is selected based on the GEBV. Therefore, the top TP update method will not always select the same individuals. The oracle TP update method also results in a training population with a high genetic relationship w.r.t. the breeding population, indicating the importance of maximizing the genetic relationship.

The residual error is the absolute difference between the genetic value and the phenotypic value of an individual. Over the first breeding cycles, the oracle TP update method selects individuals with a low residual error. *In vivo*, the residual error is unknown and thus selecting individuals that minimize the residual error cannot easily be achieved. The PEVmean update method (Rincent et al., 2012) selects individuals by minimizing specific contrasts of the prediction error variance matrix that is associated with the random effects part of the mixed model equations, but according to Neyhart et al. (2017) it was not able to outperform other update methods in the long term. This is also confirmed in **Figure 3**. Therefore, non-oracle update methods will probably not be able to reach the same long-term genetic values as the oracle TP update method as long as the residual error cannot be measured or predicted more accurately.

The two driving forces of the oracle TP update method are the maximization of the genetic relationship between the TP and the breeding population and the minimization of the residual error of the TP. Although the genetic relationship can easily be maximized, minimizing the residual error may be more difficult. High-throughput phenotyping could result in data of higher quality, thus reducing the residual error. In this simulation study, the oracle TP update method only required a TP size of approximately 150 individuals. This indicates that phenotyping efforts should probably focus more on quality than on quantity.

## 5 Future prospects

A parental selection method should aim to maximize the fixation of favorable QTL alleles. However, if the reliability of the GEBVs is low, this may also result in premature convergence of the genetic value. Therefore, a parental selection method should also aim to preserve genetic variation to the highest extent possible. Combined with high genetic progress, this will result in high genetic gains while avoiding a premature convergence of the genetic value.

**Figure 9:**
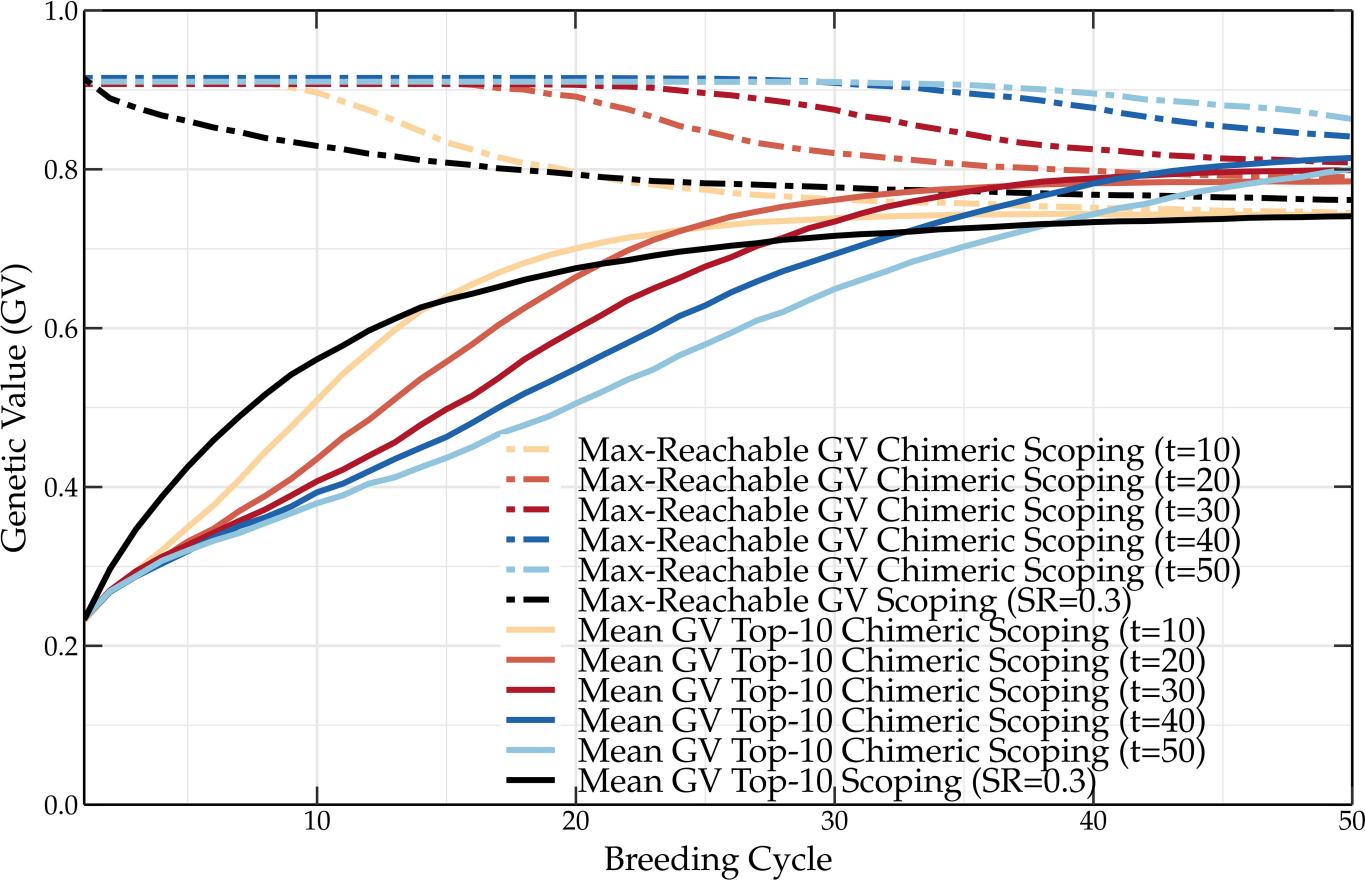
Mean genetic value of the top-10 individuals and maximum reachable genetic value of a breeding population using the scoping method and the chimeric scoping method. Compared to the scoping method, chimeric scoping results in lower short-term but higher long-term genetic values.

In one way, both the scoping and deep scoping methods maximize the genetic gain while preserving a certain amount of genetic variation in the parental population. The preservation of genetic variation can at a later stage be reduced to fully maximize the genetic gain. This was demonstrated with the adaptive scoping method (Vanavermaete et al., 2022), but this idea can be further improved upon by dividing the crossing block design into two parts. Assuming a parental selection of 100 parents that are paired into 50 couples, the first N couples are selected according to the scoping method. The remaining 50−N couples are selected solely using the F-score to select both parents, fully maximizing the genetic variation of the parental population. This will result in offspring with a lower genetic value but a broad genetic variation. Because P_1_ parents are still selected via truncation selection in the scoping method, increasing N will result in higher short-, but lower long-term genetic values. Therefore, in due time, by increasing the number of parental pairs that are selected according to the scoping method, the genetic gain will gradually increase. This concept, coined *chimeric scoping*, is shown in **Figure 9**. Just like a chimera is composed out of cells with more than one distinct phenotype, the crossing block design of chimeric scoping consists of two separate parental selection strategies. By preserving the genetic variation till the t -th breeding cycle, higher long-term genetic values of up to 10 percentage points are observed compared to the original scoping method. This demonstrates that maximizing the genetic progress while still preserving genetic variation can result in higher genetic values in the long term. We believe that this approach can be used to further develop new parental selection methods.

## 6 Conclusion

The results obtained by the oracle parental selection method indicate that current methods to select the parental population are far from optimal. Although the scoping method increases the long-term genetic values considerably compared to truncation selection, the optimal breeding strategy has not yet been found, incentivizing the quest for more performant methods. Similarly, the TP update methods are also not able to maximize the genetic gain compared to the oracle TP update method. Although the oracle method clearly adds individuals to the TP that maximize the genetic relationship between the TP and the breeding population, it also selects individuals with a lower residual error, which cannot easily be achieved *in vivo*. Therefore, TP update methods will probably never be able to reach the same long-term genetic values as the oracle TP update method does. Nevertheless, this also shows that phenotyping technology should perhaps focus more on quality and less on quantity.

## Data availability statement

The datasets presented in this study can be found in online repositories. The names of the repository/repositories and accession number(s) can be found below: https://github.com/biointec/OracleSelection.

## Author contributions

DV, SM, JF, and BDB conceived and supervised the study. DV designed and performed the experiments, and wrote an early version of the manuscript. All authors contributed to the article and approved the submitted version.
