# Supplementary material for: Oracle selection provides insight into how far off practice is from Utopia in plant breeding
Source: Front Plant Sci. 2023 Jul 21;14:1218665. doi: 10.3389/fpls.2023.1218665 (PMC10401442; doi:10.3389/fpls.2023.1218665)
Supplement: Supplementary file 1 [file DataSheet_1.pdf]

frontiers

Supplementary Material

Table S1. The mean genetic value and the standard deviation of the top-10 individuals for truncation selection, the scoping (SR=0.3), deep scoping (BC=5), OCS (SR=0.3), and oracle selection methods over 100 experiments.

Cycle	Truncation Selection	Scoping (SR=0.3)	Deep Scoping (BC=5)	OCS (SR=0.3)	Oracle Selection
1	0.23 ± 0.08	0.24 ± 0.09	-	0.23 ± 0.09	0.23 ± 0.09
3	0.34 ± 0.08	0.35 ± 0.09	-	0.34 ± 0.08	0.36 ± 0.09
5	0.41 ± 0.09	0.43 ± 0.08	0.41 ± 0.09	0.41 ± 0.07	0.47 ± 0.09
7	0.46 ± 0.08	0.49 ± 0.08	0.46 ± 0.09	0.46 ± 0.08	0.56 ± 0.08
9	0.50 ± 0.08	0.54 ± 0.07	0.50 ± 0.08	0.50 ± 0.08	0.64 ± 0.08
11	0.52 ± 0.08	0.58 ± 0.07	0.53 ± 0.08	0.53 ± 0.08	0.70 ± 0.07
13	0.54 ± 0.08	0.61 ± 0.07	0.56 ± 0.08	0.55 ± 0.08	0.75 ± 0.07
15	0.55 ± 0.08	0.64 ± 0.07	0.58 ± 0.08	0.56 ± 0.08	0.78 ± 0.07
17	0.55 ± 0.08	0.66 ± 0.07	0.60 ± 0.08	0.56 ± 0.08	0.80 ± 0.06
19	0.56 ± 0.08	0.67 ± 0.07	0.62 ± 0.08	0.57 ± 0.08	0.82 ± 0.06
21	0.56 ± 0.08	0.68 ± 0.07	0.63 ± 0.08	0.57 ± 0.08	0.83 ± 0.06
23	0.56 ± 0.08	0.69 ± 0.07	0.65 ± 0.08	0.57 ± 0.08	0.84 ± 0.06
25	0.56 ± 0.08	0.70 ± 0.07	0.66 ± 0.08	0.57 ± 0.08	0.85 ± 0.06
27	0.56 ± 0.08	0.71 ± 0.07	0.67 ± 0.08	0.57 ± 0.08	0.85 ± 0.06
29	0.56 ± 0.08	0.72 ± 0.07	0.68 ± 0.07	0.57 ± 0.08	0.86 ± 0.06
31	0.56 ± 0.08	0.72 ± 0.07	0.69 ± 0.07	0.57 ± 0.08	0.86 ± 0.06
33	0.56 ± 0.08	0.73 ± 0.07	0.69 ± 0.07	0.56 ± 0.08	0.87 ± 0.06
35	0.56 ± 0.08	0.73 ± 0.07	0.70 ± 0.07	0.56 ± 0.08	0.87 ± 0.06
37	0.56 ± 0.08	0.73 ± 0.07	0.71 ± 0.06	0.56 ± 0.08	0.87 ± 0.06
39	0.56 ± 0.08	0.74 ± 0.07	0.71 ± 0.06	0.56 ± 0.08	0.87 ± 0.06
41	0.56 ± 0.08	0.74 ± 0.07	0.72 ± 0.06	0.56 ± 0.08	0.87 ± 0.06
43	0.56 ± 0.08	0.74 ± 0.07	0.72 ± 0.06	0.56 ± 0.08	0.87 ± 0.06
45	0.56 ± 0.08	0.74 ± 0.07	0.73 ± 0.06	0.56 ± 0.08	0.88 ± 0.06
47	0.56 ± 0.08	0.74 ± 0.07	0.73 ± 0.06	0.56 ± 0.08	0.88 ± 0.06
49	0.56 ± 0.08	0.74 ± 0.07	0.74 ± 0.06	0.56 ± 0.08	0.88 ± 0.06
50	0.56 ± 0.08	0.74 ± 0.07	0.74 ± 0.06	0.56 ± 0.08	0.88 ± 0.06

Table S2. The maximum reachable genetic value and the standard deviation of the top-10 individuals for truncation selection, the scoping (SR=0.3), deep scoping (BC=5), OCS (SR=0.3), and oracle selection methods over 100 experiments.

Cycle	Truncation Selection	Scoping (SR=0.3)	Deep Scoping (BC=5)	OCS (SR=0.3)	Oracle Selection
1	0.91 ± 0.04	0.92 ± 0.04	-	0.92 ± 0.05	0.91 ± 0.05
3	0.78 ± 0.07	0.88 ± 0.06	-	0.80 ± 0.08	0.91 ± 0.05
5	0.72 ± 0.08	0.86 ± 0.06	0.73 ± 0.08	0.73 ± 0.08	0.91 ± 0.05
7	0.68 ± 0.08	0.85 ± 0.06	0.84 ± 0.07	0.69 ± 0.07	0.91 ± 0.05
9	0.65 ± 0.09	0.84 ± 0.06	0.86 ± 0.07	0.66 ± 0.07	0.91 ± 0.05
11	0.63 ± 0.09	0.83 ± 0.07	0.87 ± 0.06	0.65 ± 0.07	0.91 ± 0.05
13	0.62 ± 0.09	0.82 ± 0.07	0.86 ± 0.07	0.63 ± 0.07	0.91 ± 0.05
15	0.6 ± 0.09	0.81 ± 0.07	0.87 ± 0.07	0.62 ± 0.08	0.91 ± 0.05
17	0.59 ± 0.08	0.81 ± 0.07	0.87 ± 0.07	0.61 ± 0.08	0.91 ± 0.05
19	0.59 ± 0.08	0.80 ± 0.07	0.88 ± 0.06	0.60 ± 0.08	0.91 ± 0.05
21	0.58 ± 0.08	0.80 ± 0.07	0.87 ± 0.06	0.60 ± 0.08	0.91 ± 0.05
23	0.58 ± 0.08	0.79 ± 0.07	0.87 ± 0.06	0.59 ± 0.08	0.91 ± 0.05
25	0.58 ± 0.09	0.79 ± 0.07	0.87 ± 0.06	0.59 ± 0.08	0.91 ± 0.05
27	0.57 ± 0.09	0.78 ± 0.07	0.87 ± 0.06	0.58 ± 0.08	0.91 ± 0.05
29	0.57 ± 0.09	0.78 ± 0.07	0.87 ± 0.06	0.58 ± 0.08	0.91 ± 0.05
31	0.57 ± 0.09	0.78 ± 0.07	0.88 ± 0.06	0.57 ± 0.08	0.91 ± 0.05
33	0.57 ± 0.09	0.78 ± 0.07	0.88 ± 0.06	0.57 ± 0.08	0.91 ± 0.05
35	0.57 ± 0.09	0.77 ± 0.07	0.88 ± 0.06	0.57 ± 0.08	0.91 ± 0.05
37	0.57 ± 0.09	0.77 ± 0.07	0.88 ± 0.06	0.57 ± 0.08	0.91 ± 0.05
39	0.57 ± 0.08	0.77 ± 0.07	0.88 ± 0.06	0.56 ± 0.08	0.91 ± 0.05
41	0.56 ± 0.08	0.77 ± 0.07	0.88 ± 0.06	0.56 ± 0.08	0.91 ± 0.05
43	0.56 ± 0.09	0.77 ± 0.07	0.88 ± 0.06	0.56 ± 0.08	0.91 ± 0.05
45	0.56 ± 0.09	0.77 ± 0.07	0.88 ± 0.06	0.56 ± 0.08	0.91 ± 0.05
47	0.56 ± 0.08	0.77 ± 0.07	0.88 ± 0.06	0.56 ± 0.08	0.91 ± 0.05
49	0.56 ± 0.08	0.76 ± 0.07	0.89 ± 0.06	0.56 ± 0.08	0.91 ± 0.05
50	0.56 ± 0.08	0.76 ± 0.07	0.89 ± 0.06	0.56 ± 0.08	0.91 ± 0.05

Table S3. The mean genetic value and the standard deviation of the top-10 individuals using truncation selection and updating the TP according to the top, tails, random, CDmean, PEVmean, TrainSel and oracle TP selection methods over 100 experiments.

Cycle	Top	Tails	Random	CDmean	PEVmean	TrainSel	Oracle
1	0.23 ± 0.09	0.24 ± 0.09	0.23 ± 0.09	0.23 ± 0.09	0.23 ± 0.09	0.24 ± 0.09	0.23 ± 0.09
2	0.30 ± 0.08	0.30 ± 0.08	0.30 ± 0.08	0.30 ± 0.09	0.30 ± 0.09	0.30 ± 0.09	0.30 ± 0.09
3	0.35 ± 0.08	0.34 ± 0.08	0.34 ± 0.08	0.34 ± 0.09	0.35 ± 0.08	0.35 ± 0.08	0.37 ± 0.08
4	0.38 ± 0.08	0.38 ± 0.08	0.38 ± 0.08	0.38 ± 0.09	0.38 ± 0.08	0.39 ± 0.09	0.42 ± 0.08
5	0.42 ± 0.08	0.41 ± 0.08	0.41 ± 0.08	0.41 ± 0.09	0.41 ± 0.08	0.42 ± 0.08	0.48 ± 0.07
6	0.44 ± 0.08	0.43 ± 0.08	0.44 ± 0.08	0.43 ± 0.09	0.44 ± 0.08	0.44 ± 0.09	0.52 ± 0.07
7	0.47 ± 0.08	0.45 ± 0.08	0.45 ± 0.08	0.45 ± 0.08	0.46 ± 0.08	0.47 ± 0.09	0.55 ± 0.07
8	0.48 ± 0.08	0.47 ± 0.09	0.47 ± 0.08	0.46 ± 0.09	0.48 ± 0.08	0.48 ± 0.09	0.58 ± 0.07
9	0.50 ± 0.08	0.49 ± 0.09	0.49 ± 0.08	0.47 ± 0.09	0.49 ± 0.08	0.50 ± 0.09	0.60 ± 0.07
10	0.51 ± 0.09	0.50 ± 0.09	0.50 ± 0.08	0.49 ± 0.09	0.50 ± 0.08	0.51 ± 0.09	0.62 ± 0.08
11	0.51 ± 0.09	0.50 ± 0.09	0.51 ± 0.08	0.50 ± 0.09	0.51 ± 0.08	0.52 ± 0.09	0.64 ± 0.08
12	0.52 ± 0.09	0.51 ± 0.09	0.51 ± 0.08	0.51 ± 0.09	0.52 ± 0.08	0.53 ± 0.09	0.65 ± 0.08
13	0.53 ± 0.09	0.51 ± 0.09	0.52 ± 0.08	0.51 ± 0.09	0.52 ± 0.08	0.53 ± 0.09	0.65 ± 0.08
14	0.53 ± 0.09	0.52 ± 0.09	0.52 ± 0.08	0.52 ± 0.09	0.53 ± 0.08	0.54 ± 0.09	0.66 ± 0.08
15	0.53 ± 0.09	0.52 ± 0.09	0.53 ± 0.08	0.52 ± 0.09	0.53 ± 0.08	0.54 ± 0.09	0.66 ± 0.08

Table S4. The maximum reachable genetic value and the standard deviation of the top-10 individuals using truncation selection and updating the TP according to the top, tails, random, CDmean, PEVmean, TrainSel and oracle TP selection methods over 100 experiments.

Cycle	Top	Tails	Random	CDmean	PEVmean	TrainSel	Oracle
1	0.91 ± 0.05	0.91 ± 0.05	0.92 ± 0.05	0.92 ± 0.04	0.91 ± 0.05	0.92 ± 0.05	0.92 ± 0.05
2	0.85 ± 0.07	0.84 ± 0.07	0.85 ± 0.07	0.85 ± 0.07	0.84 ± 0.06	0.85 ± 0.06	0.84 ± 0.06
3	0.80 ± 0.08	0.79 ± 0.08	0.81 ± 0.08	0.81 ± 0.07	0.79 ± 0.07	0.80 ± 0.07	0.80 ± 0.08
4	0.76 ± 0.09	0.76 ± 0.08	0.77 ± 0.08	0.76 ± 0.08	0.76 ± 0.08	0.77 ± 0.08	0.77 ± 0.08
5	0.74 ± 0.09	0.73 ± 0.09	0.74 ± 0.09	0.74 ± 0.08	0.73 ± 0.08	0.75 ± 0.09	0.75 ± 0.09
6	0.71 ± 0.09	0.71 ± 0.09	0.72 ± 0.09	0.71 ± 0.08	0.70 ± 0.08	0.73 ± 0.09	0.73 ± 0.09
7	0.69 ± 0.09	0.68 ± 0.09	0.69 ± 0.09	0.69 ± 0.08	0.69 ± 0.08	0.71 ± 0.09	0.72 ± 0.09
8	0.67 ± 0.09	0.67 ± 0.09	0.67 ± 0.09	0.67 ± 0.08	0.67 ± 0.08	0.69 ± 0.09	0.70 ± 0.09
9	0.65 ± 0.09	0.65 ± 0.09	0.66 ± 0.09	0.66 ± 0.08	0.66 ± 0.08	0.67 ± 0.09	0.69 ± 0.09
10	0.64 ± 0.10	0.64 ± 0.09	0.65 ± 0.10	0.65 ± 0.08	0.65 ± 0.08	0.66 ± 0.09	0.69 ± 0.09
11	0.63 ± 0.10	0.63 ± 0.09	0.64 ± 0.09	0.63 ± 0.08	0.64 ± 0.08	0.65 ± 0.09	0.68 ± 0.09
12	0.62 ± 0.10	0.62 ± 0.09	0.63 ± 0.09	0.62 ± 0.09	0.63 ± 0.08	0.64 ± 0.09	0.68 ± 0.09
13	0.61 ± 0.09	0.61 ± 0.09	0.62 ± 0.09	0.61 ± 0.08	0.62 ± 0.09	0.63 ± 0.09	0.67 ± 0.09
14	0.60 ± 0.10	0.61 ± 0.09	0.61 ± 0.09	0.61 ± 0.09	0.61 ± 0.09	0.62 ± 0.09	0.67 ± 0.08
15	0.60 ± 0.10	0.60 ± 0.09	0.60 ± 0.09	0.60 ± 0.09	0.60 ± 0.09	0.61 ± 0.09	0.67 ± 0.08

Table S5. **The mean genetic value and the standard deviation of the top-10 individuals using the scoping method (SR=0.3) and updating the TP according to the top, tails, random, CDmean, PEVmean, TrainSel and oracle TP selection methods over 100 experiments.**

Cycle	Top	Tails	Random	CDmean	PEVmean	TrainSel	Oracle
1	0.23 ± 0.09	0.23 ± 0.09	0.23 ± 0.09	0.23 ± 0.09	0.23 ± 0.09	0.23 ± 0.09	0.23 ± 0.09
2	0.30 ± 0.09	0.30 ± 0.08	0.29 ± 0.09	0.29 ± 0.09	0.30 ± 0.09	0.30 ± 0.08	0.30 ± 0.09
3	0.34 ± 0.08	0.34 ± 0.08	0.34 ± 0.08	0.34 ± 0.09	0.34 ± 0.08	0.35 ± 0.08	0.36 ± 0.08
4	0.38 ± 0.08	0.38 ± 0.08	0.38 ± 0.08	0.38 ± 0.08	0.38 ± 0.08	0.38 ± 0.08	0.42 ± 0.08
5	0.41 ± 0.08	0.41 ± 0.08	0.41 ± 0.08	0.41 ± 0.08	0.41 ± 0.08	0.41 ± 0.08	0.47 ± 0.07
6	0.43 ± 0.08	0.43 ± 0.08	0.43 ± 0.07	0.43 ± 0.08	0.44 ± 0.08	0.44 ± 0.08	0.52 ± 0.07
7	0.45 ± 0.08	0.46 ± 0.08	0.46 ± 0.08	0.45 ± 0.08	0.46 ± 0.08	0.47 ± 0.09	0.56 ± 0.07
8	0.47 ± 0.08	0.48 ± 0.08	0.47 ± 0.07	0.47 ± 0.08	0.48 ± 0.08	0.49 ± 0.09	0.60 ± 0.07
9	0.49 ± 0.08	0.49 ± 0.09	0.49 ± 0.08	0.49 ± 0.08	0.49 ± 0.08	0.51 ± 0.09	0.63 ± 0.07
10	0.50 ± 0.08	0.51 ± 0.09	0.51 ± 0.08	0.50 ± 0.08	0.51 ± 0.08	0.53 ± 0.09	0.66 ± 0.06
11	0.52 ± 0.09	0.52 ± 0.09	0.52 ± 0.07	0.51 ± 0.08	0.52 ± 0.08	0.55 ± 0.09	0.68 ± 0.06
12	0.53 ± 0.08	0.53 ± 0.09	0.53 ± 0.07	0.53 ± 0.08	0.53 ± 0.08	0.56 ± 0.09	0.71 ± 0.07
13	0.54 ± 0.09	0.55 ± 0.09	0.54 ± 0.07	0.54 ± 0.08	0.54 ± 0.08	0.57 ± 0.09	0.72 ± 0.07
14	0.55 ± 0.09	0.56 ± 0.09	0.55 ± 0.07	0.55 ± 0.08	0.55 ± 0.08	0.59 ± 0.09	0.73 ± 0.07
15	0.56 ± 0.08	0.57 ± 0.09	0.56 ± 0.08	0.55 ± 0.08	0.56 ± 0.08	0.60 ± 0.09	0.75 ± 0.07

Table S6. The maximum reachable genetic value and the standard deviation of the top-10 individuals using the scoping method (SR=0.3) and updating the TP according to the top, tails, random, CDmean, PEVmean, TrainSel and oracle TP selection methods over 100 experiments.

Cycle	Top	Tails	Random	CDmean	PEVmean	TrainSel	Oracle
1	0.91 ± 0.05	0.91 ± 0.05	0.91 ± 0.05	0.91 ± 0.06	0.91 ± 0.05	0.92 ± 0.05	0.91 ± 0.05
2	0.89 ± 0.05	0.89 ± 0.06	0.88 ± 0.06	0.88 ± 0.06	0.89 ± 0.06	0.90 ± 0.05	0.88 ± 0.06
3	0.87 ± 0.05	0.87 ± 0.06	0.87 ± 0.07	0.87 ± 0.06	0.88 ± 0.06	0.89 ± 0.05	0.87 ± 0.07
4	0.87 ± 0.06	0.87 ± 0.07	0.87 ± 0.07	0.85 ± 0.06	0.87 ± 0.06	0.88 ± 0.05	0.86 ± 0.07
5	0.85 ± 0.06	0.86 ± 0.07	0.86 ± 0.07	0.85 ± 0.06	0.86 ± 0.06	0.86 ± 0.06	0.85 ± 0.07
6	0.85 ± 0.06	0.85 ± 0.07	0.85 ± 0.07	0.84 ± 0.07	0.86 ± 0.06	0.86 ± 0.06	0.85 ± 0.07
7	0.84 ± 0.06	0.84 ± 0.07	0.84 ± 0.07	0.83 ± 0.07	0.85 ± 0.07	0.85 ± 0.06	0.84 ± 0.07
8	0.83 ± 0.06	0.83 ± 0.07	0.83 ± 0.07	0.82 ± 0.07	0.84 ± 0.07	0.84 ± 0.06	0.83 ± 0.07
9	0.82 ± 0.07	0.83 ± 0.07	0.82 ± 0.07	0.82 ± 0.07	0.83 ± 0.07	0.83 ± 0.07	0.83 ± 0.07
10	0.81 ± 0.07	0.82 ± 0.07	0.81 ± 0.07	0.81 ± 0.07	0.82 ± 0.07	0.83 ± 0.07	0.83 ± 0.07
11	0.80 ± 0.07	0.81 ± 0.07	0.81 ± 0.07	0.81 ± 0.07	0.82 ± 0.07	0.82 ± 0.08	0.83 ± 0.07
12	0.80 ± 0.07	0.81 ± 0.07	0.80 ± 0.07	0.80 ± 0.07	0.81 ± 0.07	0.81 ± 0.08	0.82 ± 0.07
13	0.79 ± 0.07	0.80 ± 0.07	0.80 ± 0.07	0.80 ± 0.07	0.81 ± 0.07	0.81 ± 0.08	0.82 ± 0.07
14	0.78 ± 0.07	0.80 ± 0.07	0.79 ± 0.08	0.79 ± 0.07	0.80 ± 0.07	0.80 ± 0.08	0.81 ± 0.07
15	0.78 ± 0.07	0.79 ± 0.07	0.79 ± 0.08	0.78 ± 0.07	0.80 ± 0.07	0.80 ± 0.08	0.81 ± 0.07
